# Elicitation of the *in vitro* Cultures of Selected Varieties of *Vigna radiata* L. With Zinc Oxide and Copper Oxide Nanoparticles for Enhanced Phytochemicals Production

**DOI:** 10.3389/fpls.2022.908532

**Published:** 2022-07-26

**Authors:** Zunera Iqbal, Sumera Javad, Shagufta Naz, Anis Ali Shah, Adnan Noor Shah, Bilal Ahmad Paray, Aneela Gulnaz, Nader R. Abdelsalam

**Affiliations:** ^1^Department of Botany, Lahore College for Women University, Lahore, Pakistan; ^2^Department of Biotechnology, Lahore College for Women University, Lahore, Pakistan; ^3^Division of Science and Technology, Department of Botany, University of Education, Lahore, Pakistan; ^4^Department of Agricultural Engineering, Khwaja Fareed University of Engineering and Information Technology, Rahim Yar Khan, Pakistan; ^5^Department of Zoology, College of Science, King Saud University, Riyadh, Saudi Arabia; ^6^College of Pharmacy, Woosuk University, Wanju-gun, South Korea; ^7^Department of Agricultural Botany, Faculty of Agriculture, Saba Basha, Alexandria University, Alexandria, Egypt

**Keywords:** callus, glycosides, leaf explants, mung bean, nanoparticles, phenolics

## Abstract

This study was conducted to develop a protocol for *in vitro* shoot multiplication and callus induction of various mung bean varieties to obtain enhanced phytochemical content with the help of elicitors. For shoot multiplication, two types of explants (shoot tips and nodal tips) of three varieties of mung bean (Mung NCM-13, MgAT-7, and MgAT-4) were used. Both types of explants from *in vitro* and *in vivo* sources were cultured on the MS medium supplemented with different concentrations (0.25–3.0 mg/L, increment of 0.5 mg/L) and combinations of BAP and IBA as independent treatments. For callus induction, leaf explants (*in vitro* source) were cultured on MS medium supplemented with 2,4-D (1–3 mg/L) alone or in combination with BAP or NAA (0.5 and 1.0 mg/L). For the enhanced production of phenolics and glycosides, calli were cultured on MS media supplemented with zinc oxide (0.5 mg/L) and copper oxide nanoparticles (0.5 mg/L) as nano-elicitors. Results showed that *in vitro* explants responded better in terms of shoot length, number of shoots, and number of leaves per explant when compared to *in vivo* explants. Moreover, shoot tips were better than nodal explants to *in vitro* culturing parameters. All three varieties showed the optimized results in the MS medium supplemented with 1 mg/L BAP, while roots were produced only in cultures fortified with 1 mg/L IBA. The leaf explants of *in vitro* and soil-grown plantlets showed a maximum callogenic response of 90 and 80%, respectively, on MS medium supplemented with 2,4-D (3 mg/ml). Maximum phenolic content (101.4 μg of gallic acid equivalent/g) and glycoside content (34 mg of amygdalin equivalent/g of plant material) was observed in the calli cultured on MS medium supplemented with 3 mg/L of 2,4-D. Furthermore, the addition of zinc oxide (0.5 mg/L) and copper oxide (0.5 mg/L) nanoparticles to the callus culture medium significantly enhanced the phenolic content of Mung NCM-13 (26%), MgAT-7 (25.6%), and MgAT-4 (22.7%). Glycosidic content was also found to be increased in Mung NCM-13 (50%), MgAT-7 (37.5%), and MgAT-4 (25%) varieties when compared to the control. It is suggested that elicitation of *in vitro* cultures of mung beans with nanoparticles could be an effective strategy for the enhanced production of secondary metabolites.

## Introduction

Plants use various processes for their survival in biotic and abiotic stress conditions, one of the mechanisms being the production of secondary metabolites. Elicitation is the process that is used for increasing secondary metabolite production in plants (Patel and Krishnamurthy, 2013; Ahmad et al., 2020) and to increase their capacity to enhance secondary metabolite production. Elicitors are substances that cause biological and physiological changes in a plant. Plants respond to these stressors/elicitors by activating a series of processes, such as defense responses to pathogens or natural stimuli that affect the body of plants, finally improving the synthesis of phytochemicals. There are two main types of elicitors: exogenous and endogenous. Exogenous elicitors are produced by some external source like some pathogens, and endogenous elicitors are produced by plants themselves to activate their immune response to combat certain stress factors. Elicitors may be biotic, abiotic, chemical, or physical in nature (Vasconsuelo and Boland, 2007). Abiotic factors may act as elicitors or may mediate the action of other elicitors to increase the production of secondary metabolites, such as anthocyanins, flavonoids, and glycosides (Glabgen et al., 1998; Carvalho et al., 2020; Raghib et al., 2020). Salt stress is also known to play the same role (Mbarki et al., 2018; Cirillo et al., 2021; Faizan et al., 2021). Understanding of signaling pathways underlying the natural response of plants to pathogen infection has helped researchers to discover various synthetic and natural compounds that show similar responses and are called elicitors (Gomez-Vasquez et al., 2004). Recently, the successful use of nanoparticles as elicitors has been reported for the synthesis of secondary metabolites in the plant cell cultures (Rivero-Montejo et al., 2021). Elicitors show their effect by binding to elicitor-binding sites on membranes, and transmembrane protein kinase is one of them. Nanoparticles when acting as nano-elicitors are known to either bind to these elicitor-binding sites or can produce the endogenous messenger molecules that can bind with elicitor-binding sites. This may trigger an active exchange of ions across the membrane, including the influx of calcium ions, which ultimately affects the ATPase activity and increases the cytoplasmic acidity. This increased acidity further leads to the production of secondary metabolites (Khan et al., 2021). Nano-elicitors are also known to elicit secondary metabolite production by triggering oxidative gene expression (Rivero-Montejo et al., 2021).

Nanotechnology has applications in almost every field, from material science to biotechnology. Nanomaterials are defined as substances with dimensions on the nanoscale, in the size of 1–100 nm, or with an internal structure or external structure in a nanoscale (Buzea et al., 2007). Nanoparticles (NPs) have improved features as compared to their bulk material, and therefore possess improved characteristics based on their nanosize, shape, and structure (Joshi et al., 2019). NPs have the potential to be used as active elicitors in plant biotechnology, and many studies have supported their potential role in improving the genetic expression of genes associated with the production of secondary metabolites (Vasconsuelo and Boland, 2007). NPs have also identified roles in increasing the production of primary metabolites in plants, including fatty acids, amino acids, and carbohydrates (Zhao et al., 2017). NPs initiate the accumulation of reactive oxygen species in plant cells, thus triggering the signaling pathway for secondary metabolites (Marslin et al., 2017). Hence, there is a requirement to optimize the dose of NPs to have their positive action as elicitors, rather than enhancing toxicity. Earlier increased production of lycopene by tomato plants, isovitexin by barley, and epigenin and kaempferol by celery has been reported by supplementing doses of cerium oxide, cadmium oxide, and selenium NPs, respectively (Ghanati and Bakhtiarian, 2014; Vecerova et al., 2016; Li et al., 2020).

Plant tissue cultures and cell cultures are very good targets for achieving enhanced commercial production of plant secondary metabolites. The *in vitro* cultures of plants in any form, i.e., tissue culture or cell culture, under controlled physical parameters provide a technological platform for the commercial production of natural plant products. In other words, we can obtain the secondary metabolites of plants from the root, shoot, callus, or cell suspension cultures of respective plants in enhanced concentrations when compared to *in vivo* plants by controlling their culture parameters (Espinosa-Leal et al., 2018). There is no effect of environment, season, or any pest on their controlled production. Among the various methods available for reproducing plant cell culture, callus culture is an ideal method for the possible production of healthy plants and bioactive phytochemicals. Callus can be continuously modified in cultures to regenerate whole plants or produce various secondary metabolites (Manjkhola et al., 2005; Rani et al., 2017).

Here, NPs can be utilized as an advanced abiotic elicitor, where they play a role in the enhanced production of plant secondary metabolites of commercial importance in plant cell and tissue cultures (Hatami et al., 2019). In current years, a lot of discussions has been carried out by researchers on the role of nanoparticles, as an elicitor, in promoting the expression of genes that are involved in the synthesis pathway of plant secondary metabolites (Jordan et al., 2018). Ali et al. (2019a,b) investigated the *in vitro* cultures of *Caralluma tuberculata* when elicited with Ag NPs (90 μg/L), which caused an overall decrease in plant biomass but a significant increase in the total flavonoid content, total phenols, phenylalanine amonialyase (PAL), and fresh weight of plants. Meanwhile, Fazal et al. (2019) used a combination of silver and gold nanoparticles (3:1) and NAA (naphthalene acetic acid) and discovered an increase in the total flavonoid (0.61 rutin equivalents mg/g extract) and the total phenol content (7.62 gallic acid equivalent mg/g extract) of plants grown under NP treatment. Mosavat et al. (2019) also reported that the use of zinc oxide NPs in the cultures of *Thymus* and *Zataria multiflora* enhanced the thymol and carvacrol content. Ahmad et al. (2020) recognized the effect of ZnO and CuO NPs on *in vitro* root formation, non-enzymatic antioxidant activity, and steviol glycoside (SGs) production in regenerants of Candyleaf (*Stevia rebaudiana*). When 2 mg/L of ZnO NPs and 20 mg/L of CuO NPs were used as elicitors, liquid chromatography analysis revealed an effective increase in the content of steviol glycosides, stevioside (1.28 and 1.96), and rebaudioside.

Secondary plant metabolites are products of the secondary metabolism of plants that play a key role in transmitting the natural stability of the plant, especially during the defense response (Dey, 2016; Jan et al., 2021). These metabolites are often produced by stress signals, such as pathogen attacks, environmental stresses, and nutrient deficiency. Several phytochemicals are also considered as important in human medicines, such as vaccines and drugs (Rao and Ravishankar, 2002). All plants produce secondary metabolites of various nature and specific chemistry that can be exploited at the commercial level. Mung bean (*V. radiata*) is one of these plants and is a source of both nutrients and secondary metabolites. Mung beans, a member of the family Fabaceae, contain balanced nutrients, including proteins, fibers, vitamins, and minerals, all of which have essential biological value (Singh et al., 2017a). Mung bean possesses a variety of polyphenolic compounds in large quantities. The main phenolic components of mung beans include flavonoids (1.49–1.78 mg catechin eq./ g), phenolic acids (1.81–5.97 mg rutin equivalent / g), and tannins (1.00–5.75 mg/g) (Lee et al., 2011; Shi et al., 2016; Singh et al., 2016, 2017b). The HPLC-UV examination of mung bean seeds and sprouts revealed the presence of quercetin, quercetin-3-O-glucoside, and myricetin. In comparison to mung bean seeds, the concentration of these chemicals was 22 times greater in mung bean shoots (Guo et al., 2012).

Another secondary metabolite of commercial importance that is found in mung beans is amygdalin/vitamin B17, which is chemically a cyanogenic glycoside (plant secondary metabolite). Amygdalin has numerous health benefits, including blood pressure regulation and pain relief. It also strengthens the immune system and detoxifies the body. Since the 1800s, people have used vitamin B17 for anticancer treatment (Zhou et al., 2012). Cyanide is the main component that is released in the body when amygdalin is dissolved upon digestion. It inhibits and kills the growth of cancer cells while not causing harm to the healthy cells. The intake of a small handful of mung bean sprouts daily is sufficient to prevent cancer (Kim et al., 2012; Singh et al., 2016).

To date, there is no work reported on the nano-elicitation of mung bean cultures to study its effects on the production of secondary metabolites like phenolics and glycosides. Therefore, this study aimed to establish *in vitro* cultures of mung bean varieties and increase their phytochemical production, including glycosides and phenolics, using nanoparticles as elicitors. This increased synthesis of phytochemicals following induction by nano-elicitors may be exploited for the commercial production of these compounds in the future.

## Materials and Methods

### Collection of Seeds and Explant Establishment

**Figure 1:**
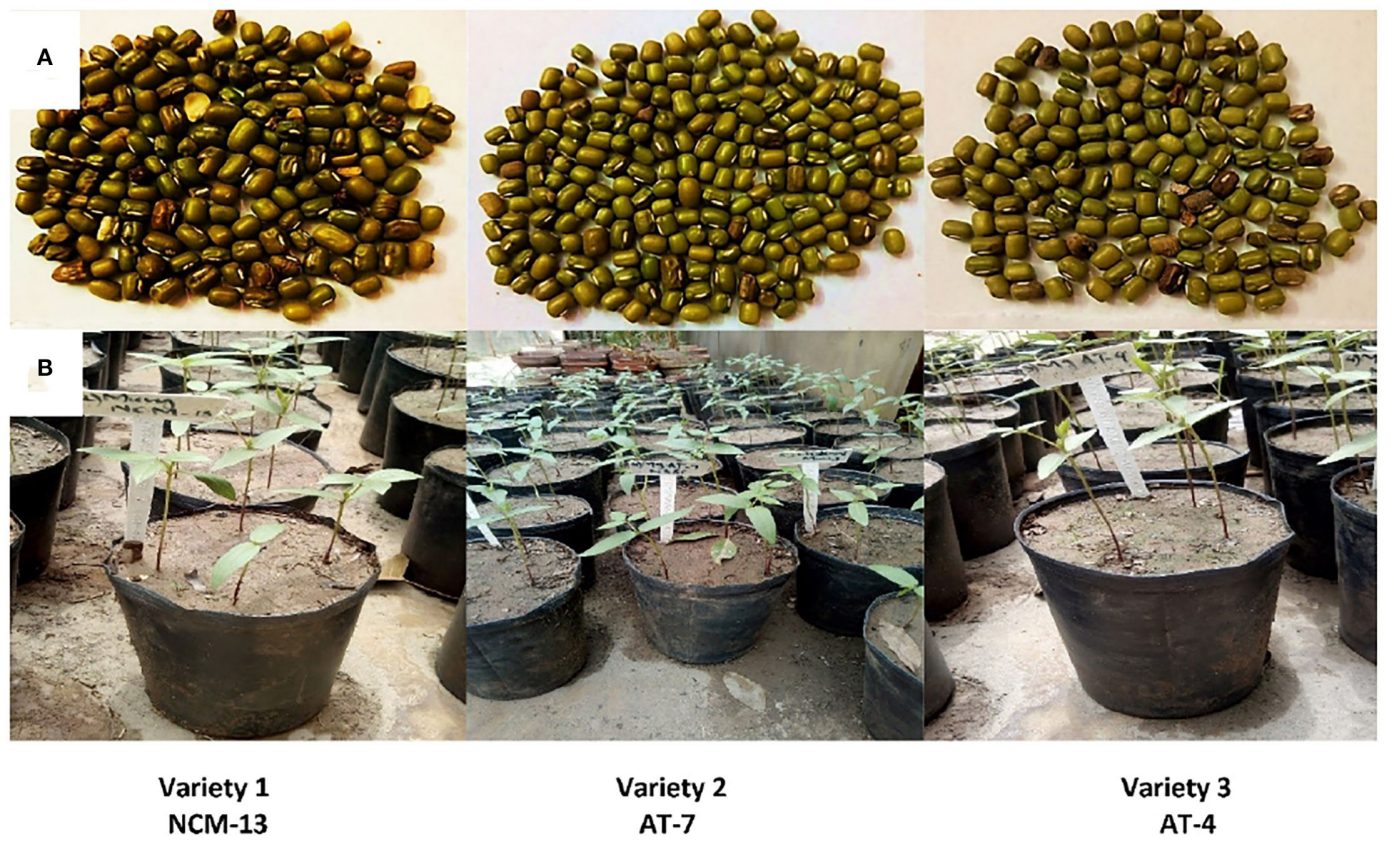
**(A)** Mung bean varieties obtained from NARC, Islamabad; **(B)** *In vivo* plants establishment of mung bean varieties.

The seeds of the varieties Mung NCM-13, MgAT-7, and MgAT-4 (Figure 1) were collected from National Agriculture Research Centre (NARC), Islamabad, Pakistan. Seeds were washed and air-dried for further use.

### *In vitro* and *in vivo* Seed Inoculation

*In vivo* plants were established to be used as explant sources, i.e., shoot and nodal tips. Seeds were then sown in loam soil (3:1, soil: sand), filled in the pots of 8 inches diameter, and replicates were set up for each variety of explant (Figure 1B). After 30 days of growth in the green house, shoot tips and nodal portions were cut, treated aseptically, and used as explants. For sterilization, soil-grown explants were washed thoroughly with running tap water and then with sterilized water. Washed explants were then dipped in 20% sodium hypochlorite solution for 10 min and cultured on a sterile medium in aseptic conditions.

For the establishment of *in vitro* explants, sterilized seeds were used. Seeds were washed with distilled water and sterilized using 70% ethanol solution for 10 min, followed by a rinse with sterile distilled water. These sterilized seeds were cultured on a basal MS medium with pH 5.7 and maintained at 24 ± 2°C with a light period of 16 h. Where seeds germinated within 1 week, shoot tips and nodal tips were aseptically removed from 14 days old cultures and were used as explants for further experimentation.

Both types of explants (*in vitro* and *in vivo*) were sub-cultured on MS medium containing different concentrations of BAP (0, 0.25, 0.5, 1, 1.5, 2.0, 2.5, and 3 mg/L) and IBA (0, 0.25, 0.5, 1, 1.5, 2.0, 2.5, and 3 mg/L). All media were autoclaved at 121°C and 15 lb for 20 min. The pH of the medium was set at 5.6. All protocols of plant tissue culture were followed for glassware and lab sterilization. Cultures were maintained at 24 ± 2°C with an 8-h dark and 16-h light periods. The shoot and nodal tips were sub-cultured regularly after every 3 weeks. Both types of explants were compared for their efficiency to initiate shooting and rooting systems.

### Callus Induction

For callus induction, immature leaves (4–7 days old) were excised from young seedlings and were cultured on MS medium containing various concentrations of MS medium + 2,4-D (1, 2.0, and 3.0 mg/L) individually and with BAP (0.5 and 1 mg/L) and NAA (0.5 and 1 mg/L). The pH of the culture medium was set at 5.6, and phytagel (1.5 g/L) was added. The medium was then autoclaved for 20 min at 121°C and 15 lb/inch^2^ for 20 min. Per treatment, 15 explants (one explant per test tube = 15 test tubes) were cultured with 3 replicates for each treatment. The temperature of the culture room was maintained at 24 ± 2°C, and a 16-h light period was maintained by using white fluorescent tubes. The calli were sub-cultured regularly after every month. Different parameters, i.e., days to initiate callus, type, color, texture of callus, and weight of callus, were recorded regularly.

#### Synthesis of Zinc Oxide and Copper Oxide Nanoparticles (Nano-Elicitors)

Zinc oxide nanoparticles were synthesized by following the protocol described by Awan et al. (2021). Aqueous extract (10 ml) of seeds of *Nigella sativa* (1 g/10 ml) was mixed with a 0.4 M solution of zinc sulfate (30 ml) in a dropwise manner. After the complete mixing of the two solutions, a color change indicated the formation of NPs. The resultant solution was centrifuged at 12,000 rpm for 10 min, and pellets were washed, dried, and preserved as ZnO NPs. For the synthesis of copper oxide nanoparticles, a slightly modified method by Prakash et al. (2018) was used. Briefly, 10 ml of copper nitrate trihydrate solution (0.1 M) was mixed dropwise with 30 ml of *N. sativa* aqueous extract (100 mg/ml) on a hot plate at 80°C. After the color change from blue to green, the mixture was centrifuged at 12,000 rpm for 10 min, and pellets were washed, dried, and preserved as NPs for future experiments. Characterization of NPs was done with the help of a UV-Visible spectrophotometer (BMS UV-2600) and a Particle Size Analyzer (BT-90 nanolaser).

#### Use of Zinc Oxide and Copper Oxide Nanoparticles in Callus Cultures as Chemical Elicitors

In the next step, callus cultures with maximum phenolic and glycoside content were selected for the nano-elicitation study. For this purpose, the callus culture medium with maximum secondary metabolite response was supplemented with ZnO NPs (0.5 mg/L) and CuO NPs (0.5 mg/L) separately. A homogenous mixture of NPs was obtained by sonication. Well-established calli were then sub-cultured on NP-supplemented media. This concentration of NPs was selected based on a screening experiment in the lab where this was found to be an optimized concentration for mung bean cultures. The concentration of NPs higher than 0.5 mg/L of MS medium caused the browning of the explants.

### Biochemical Analysis

Calli cultures and shoots with maximum biomass from the three Mung bean varieties were selected for the determination of phenolic and glycoside content.

### Phenolic Analysis

For phenolic analysis, a slightly modified method by Rebey et al. (2012) was used. Approximately, 10 ml of ethanol was taken each time for each sample. The plant material, either shoot culture or callus (1 g), was taken and macerated with solvent. The mixture was filtered after proper homogenization. Filtrate (125 μ) was mixed with 500 μl of distilled water and 125 μl of Folin-Ciocalteu reagent. The mixture was shaken and mixed well, and then 1.25 ml of Na_2_CO_3_ (7%) was added precisely. The solution was mixed properly, and the final volume was made up to 3 ml with double distilled water. It was then placed untouched in the dark for completion of the reaction between the Folin-Ciocalteu reagent and phenolics present in the plant sample. After 1.5 h, the absorption of each sample was measured on a UV-Vis spectrophotometer (BMS UV-2600) at 760 nm. Meanwhile, various concentration ranges of standard gallic acid (10–200 μg/ml) were prepared and treated with Folin-Ciocalteu reagent in the same way, and their absorbance was also noted and plotted against their concentration to make a standard curve of gallic acid. Thereafter, the total phenolic content of each plant sample was expressed as micrograms of gallic acid equivalents per gram of fresh weight (μg GAE/g) after plotting on a standard curve of gallic acid.

### Glycoside Analysis

A slightly modified method by Ilza and Pinotti (2000) was used for this purpose. One gram of plant material was macerated for 5 min with alcoholic KOH (5 ml). The macerated sample was then transferred to an aqueous solution of FeSO_4_ and FeCl_3_. This mixture was maintained at 60–70°C in the water bath for 10 min. After heating, the sample was carefully transferred to a 20% solution of HCl. At this step, the appearance of a Prussian blue color confirms the presence of the hydrogen cyanide group. This mixture was then taken in the cuvette, and its absorption was checked on a UV-Vis spectrophotometer (BMS UV-2600) at 215 nm. A standard amygdalin graph/curve was constructed and used as a reference to quantify amygdalin from the samples extracted. The cyanogenic glycoside content was measured as milligrams of amygdalin equivalents per gram of fresh plant material (mg AE/g).

### Data Evaluation

The data were collected and evaluated by one-way analysis of variance (ANOVA). Mean values were equated using statistical software for significance by Tukey's multiple range test at a 0.05% level of significance.

## Results

### Comparison of Explants

**Figure 2:**
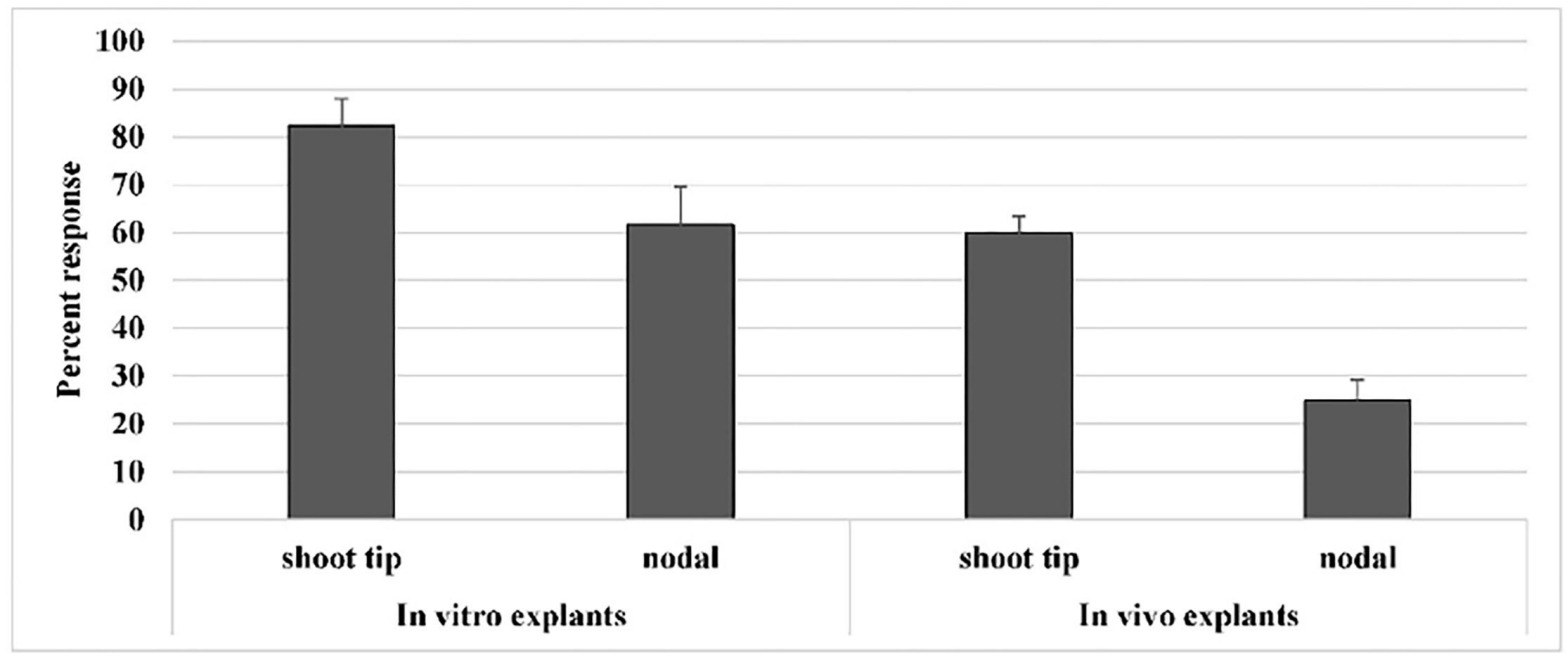
Effect of type and source of explant on *in vitro* culture establishment of varieties of mung bean.

In the present study, from *in vivo* trial, explants were obtained from 30-day-old plants of Mung NCM-13, MgAT-7, and MgAT-4 varieties and further used for *in vitro* plant establishment and callus induction (Figures 1A,B). Similarly, seeds were grown *in vitro* to get *in vitro* explants. When both types of explants were compared for their efficiency of culture establishment, it was found that *in vitro* explants provided a better source for culture establishment among all the three varieties of mung bean. A 36% increase in the response of *in vitro* shoot tip explant was observed when compared to *in vivo* shoot tip explant. Similarly, a 50% increase in response was noticed for nodal explant. Overall, shoot tip explant gave better results in both *in vivo* and *in vitro* conditions (Figure 2). The response was calculated based on the days to show the response and weight of the grown plants.

### Shoot Development of Mung Cultures

**Figure 3:**
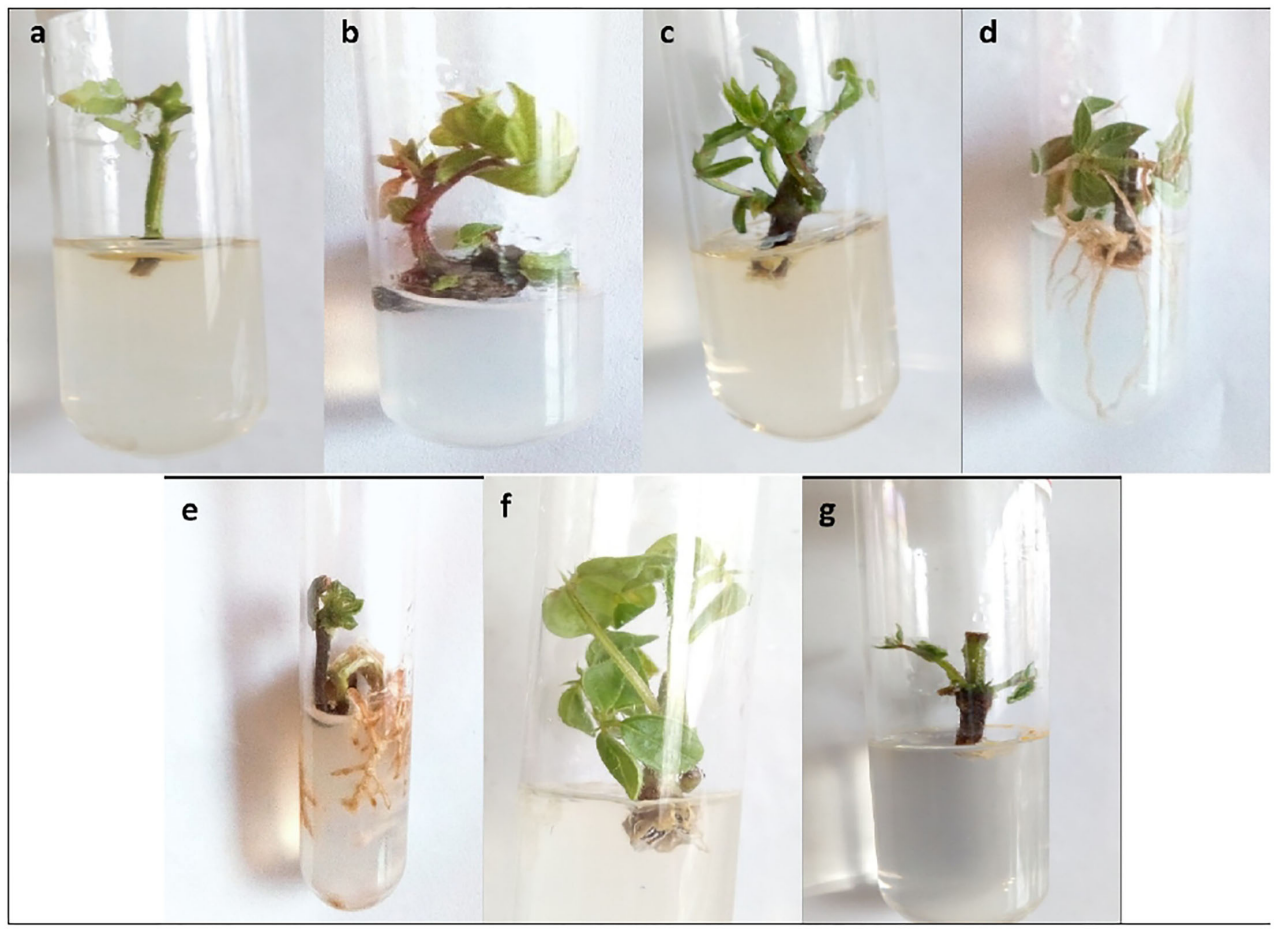
*In vitro* culture establishment of three varieties of mung bean **(a)** Variety 1 (shoot tip) in MS + BAP 1 mg/L; **(b)** Variety 2 (shoot tip) in MS + BAP 1 mg/L; **(c)** Variety 3 (shoot tip) in MS + BAP 1 mg/L; **(d)** Variety 1 (Shoot tip) in MS + IBA 1 mg/L; **(e)** Variety 2 (Shoot tip) in MS + IBA 1 mg/L; **(f)** Variety 3 (Shoot tip) in MS + IBA 1 mg/L; **(g)** Variety 1 (node) in MS + BAP 1 mg/L.

The objective of the next experiment was to find the effect of growth regulators on *in vitro* growth of mung bean varieties. The maximum shoot length (5.07 cm) of Mung NCM-13 was shown in MS+BAP (1 mg/L) medium. MS medium supplemented with 1 mg/L IBA also showed good results with a shoot length of 4.51 cm with shoot tip explant, as explained in Figure 3 and Table 1. MgAT-7 (V2) also showed the optimized results in MS+BAP 1 medium for shoot tip growth with 4.63 cm length, while IBA did not work well for this variety using any explant. The third variety of mung beans, i.e., MgAT-4 also showed better results in MS+BAP (1 mg/L) medium for shoot tip, i.e., 4.62 cm (Figure 3).

**Table 1:** Effect of various media compositions on *in vitro* culture establishment of three varieties of *Vigna radiate*.

**Type of explant**	**MS medium supplemented with**	**Concentration** **(mg/L)**	**V_1_** **Mung-NCM-13**		**V_2_** **Mung-AT7**		**V_3_** **Mung-AT4**	
			**Shoot length**	**Number of leaves**	**Shoot length**	**Number of leaves**	**Shoot length**	**Number of leaves**
Shoot tip	BAP	0.00	4.00^bcd^ ± 0.08	3.0^de^ ± 0.05	4.00^b^ ± 0.11	2.50^b^ ± 0.09	3.69^ab^ ± 0.12	2.50^d^ ± 0.07
		0.25	4.09^bc^ ± 0.10	2.8^e^ ± 0.11	3.90^bc^ ± 0.12	2.9^b^ ± 0.11	3.59^bc^ ± 0.17	3.50^c^ ± 0.19
		0.50	4.24^b^ ± 0.05	3.5^cd^ ± 0.30	3.95^b^ ± 0.09	3.5^b^ ± 0.11	3.70^ab^ ± 0.09	4.48^b^ ± 0.22
		1.00	5.07^a^ ± 0.09	6.7^a^ ± 0.15	4.63^a^ ± 0.08	5.5^a^ ± 0.05	4.62^a^ ± 0.06	7.80^a^ ± 0.28
		1.50	3.50^i^ ± 0.02	5.6^b^ ± 0.22	3.73^bcd^ ± 0.07	4.1^a^ ± 0.07	4.00^ab^ ± 0.12	6.41^a^ ± 0.31
		2.00	3.24^ij^ ± 0.05	5.6^b^ ± 0.17	3.56^bcd^ ± 0.11	3.90^ab^ ± 0.07	4.00^ab^ ± 0.19	5.32^b^ ± 0.08
		2.50	3.20^ij^ ± 0.10	3.0^bc^ ± 0.02	3.49^bcd^ ± 0.06	3.00^b^ ± 0.01	3.52^bc^ ± 0.06	5.25^b^ ± 0.12
		3.00	3.20^ij^ ± 0.12	2.8^e^ ± 0.05	3.21^def^ ± 0.03	3.00^b^ ± 0.15	3.43^bc^ ± 0.09	1.22^e^ ± 0.15
Nodal region	BAP	0.25	4.21^cd^ ± 0.08	2.9^de^ ± 0.09	3.00^fgh^ ± 0.11	2.45^b^ ± 0.19	3.59^ab^ ± 0.18	3.55^c^ ± 0.15
		0.50	4.30^d^ ± 0.09	4.0^c^ ± 0.10	3.13^efg^ ± 0.15	2.50^b^ ± 0.09	3.48^bc^ ± 0.05	4.55^b^ ± 0.05
		1.00	4.57^b^ ± 0.10	4.0^c^ ± 0.10	3.61^bcd^ ± 0.09	4.80^a^ ± 0.08	4.66^a^ ± 0.05	2.40^d^ ± 0.11
		1.50	3.50^fgh^ ± 0.09	4.0^c^ ± 0.05	3.24^def^ ± 0.07	4.00^a^ ± 0.09	4.00ab ± 0.05	2.40^d^ ± 0.08
		2.00	3.30^hij^ ± 0.08	3.9^c^ ± 0.02	3.00^fgh^ ± 0.01	3.40^b^ ± 0.11	3.50^bc^ ± 0.09	2.40^d^ ± 0.07
		2.50	3.29^hij^ ± 0.05	2.8^e^ ± 0.09	3.10^efg^ ± 0.15	2.50^b^ ± 0.09	3.49bc ± 0.19	2.40^d^ ± 0.04
		3.00	3.30^hij^ ± 0.04	2.8^e^ ± 0.03	3.00^fgh^ ± 0.07	2.50^b^ ± 0.08	3.50^bc^ ± 0.19	2.40^d^ ± 0.04
Shoot tip	IBA	0.25	4.30^cde^ ± 0.01	3.0^de^ ± 0.03	3.00^fgh^ ± 0.09	2.90^b^ ± 0.05	4.00^ab^ ± 0.21	2.50^d^ ± 0.16
		0.50	4.45^bc^ ± 0.03	4.0^de^ ± 0.1	3.00^fgh^ ± 0.09	2.90^b^ ± 0.05	4.00^ab^ ± 0.22	2.50^d^ ± 0.17
		1.00	4.51^b^ ± 0.05	5.4^de^ ± 0.09	3.20^def^ ± 0.05	2.90^b^ ± 0.05	4.13^ab^ ± 0.07	2.50^d^ ± 0.13
		1.50	3.93^cde^ ± 0.11	3.0^de^ ± 0.06	3.00^fgh^ ± 0.09	2.90^b^ ± 0.14	3.60^abc^ ± 0.14	2.40^d^ ± 0.17
		2.00	3.77^efg^ ± 0.15	2.9^de^ ± 0.02	2.69^ghi^ ± 0.09	2.50^b^ ± 0.03	3.30^bc^ ± 0.11	2.40^d^ ± 0.01
		2.50	3.30^hij^ ± 0.09	2.5^ef^ ± 0.05	2.60^ghi^ ± 0.17	2.50^b^ ± 0.09	3.20^bc^ ± 0.11	2.40^d^ ± 0.05
		3.00	3.34^hij^ ± 0.05	2.0^f^ ± 0.03	2.20^i^ ± 0.11	2.50^b^ ± 0.06	3.11^bc^ ± 0.06	2.40^d^ ± 0.05
Nodal region	IBA	0.25	4.00^cde^ ± 0.05	2.5^ef^ ± 0.08	3.00^fgh^ ± 0.05	2.40^b^ ± 0.11	4.00^ab^ ± 0.05	2.90^d^ ± 0.09
		0.50	4.00^cde^ ± 0.19	2.5^ef^ ± 0.08	3.22^dcf^ ± 0.05	2.40^b^ ± 0.09	4.09^ab^ ± 0.08	2.90^d^ ± 0.07
		1.00	4.97^a^ ± 0.12	2.5^ef^ ± 0.05	3.22^def^ ± 0.12	2.45^b^ ± 0.11	4.24ab ± 0.11	2.50^d^ ± 0.05
		1.50	3.67^efg^ ± 0.05	2.5^ef^ ± 0.03	3.30^def^ ± 0.08	2.50^b^ ± 0.11	3.30^bc^ ± 0.12	2.50^d^ ± 0.09
		2.00	3.20^ij^ ± 0.04	2.5^ef^ ± 0.05	3.95^b^ ± 0.07	2.40^b^ ± 0.17	2.69^c^ ± 0.09	2.40^d^ ± 0.09
		2.50	3.00^hij^ ± 0.11	2.5^ef^ ± 0.11	3.42^cde^ ± 0.11	2.50^b^ ± 0.07	2.60^c^ ± 0.04	2.10^de^ ± 0.06
		3.00	3.00^hij^ ± 0.12	2.5^ef^ ± 0.10	3.40^cde^ ± 0.09	2.50^b^ ± 0.12	2.60^c^ ± 0.06	2.10^de^ ± 0.05

### Number of Leaves per Explant of Mung Cultures

The number of leaves is another well-established parameter to check *in vitro* growth of explants. In the present study, leaves were also significantly affected by the type of explant and type of medium supplied in all the three mung bean varieties. An optimized number of leaves were also noticed in MS medium supplemented with 1 mg/L BAP, i.e., 6.7, 5.5, and 7.8 leaves per explant for the mung bean varieties 1, 2, and 3, respectively (Table 1).

### Rooting of Mung Cultures

The results presented in Figure 3 and Table 2 show that rooting was not established in the MS medium supplemented with 1 mg/L BAP for all the three varieties of mung bean. However, the maximum number (eight roots per explant) and the maximum length of roots (5.4 cm) were obtained in the MS medium supplemented with 1 mg/L IBA for variety 1, i.e., Mung-NCM-13. In variety 2, the maximum root length was noticed to be 1 cm with 4.9 roots per explant, which were not very strong in the same medium as V1. In variety 3, maximum root length (1.70 cm) and the number of roots (4.3 roots per explant) were also noticed in MS+ IBA (1 mg/L) medium.

**Table 2:** Effect of MS+ IBA on rooting of *in vitro* culture establishment of three varieties of *Vigna radiate*.

**Type of explant**	**MS medium supplemented with**	**Conc.** **(mg/L)**	**V_1_** **Mung-NCM-13**		**V_2_** **Mung-AT7**		**V_3_** **Mung-AT4**	
			**Root length (cm)**	**Number of roots**	**Root length (cm)**	**Number of roots**	**Root length (cm)**	**Number of roots**
Shoot tip	IBA	0.25	0.0^e^ ± 0.00	0.0^e^ ± 0.00	0.0^c^ ± 0.00	0.0^e^ ± 0.00	0.0^d^ ± 0.00	0.0^e^ ± 0.00
		0.50	2.1^c^ ± 0.01	3.0^c^ ± 0.09	1.00^a^ ± 0.00	2.76^c^ ± 0.09	1.00^c^ ± 0.00	2.10^c^ ± 0.00
		1.00	5.2^a^ ± 0.03	8.0^a^ ± 0.01	0.20^c^ ± 0.01	4.90^a^ ± 0.15	1.13^c^ ± 0.01	4.30^a^ ± 0.00
		1.50	4.3^b^ ± 0.01	4.6^b^ ± 0.10	0.30^b^ ± 0.01	3.23^b^ ± 0.09	1.64^a^ ± 0.03	3.33^b^ ± 0.00
		2.00	1.1^d^ ± 0.05	2.9^c^ ± 0.05	0.09^b^ ± 0.02	2.50^c^ ± 0.08	1.70^a^ ± 0.01	1.71^d^ ± 0.00
		2.50	1.1^d^ ± 0.01	1.5^d^ ± 0.05	0.10^b^ ± 0.00	2.10^c^ ± 0.08	1.52^b^ ± 0.02	1.12^d^ ± 0.00
		3.00	1.0^d^ ± 0.01	1.0^d^ ± 0.01	0.20^b^ ± 0.00	1.00^d^ ± 0.00	1.12^c^ ± 0.06	1.00^d^ ± 0.00

### Callus Induction

**Figure 4:**
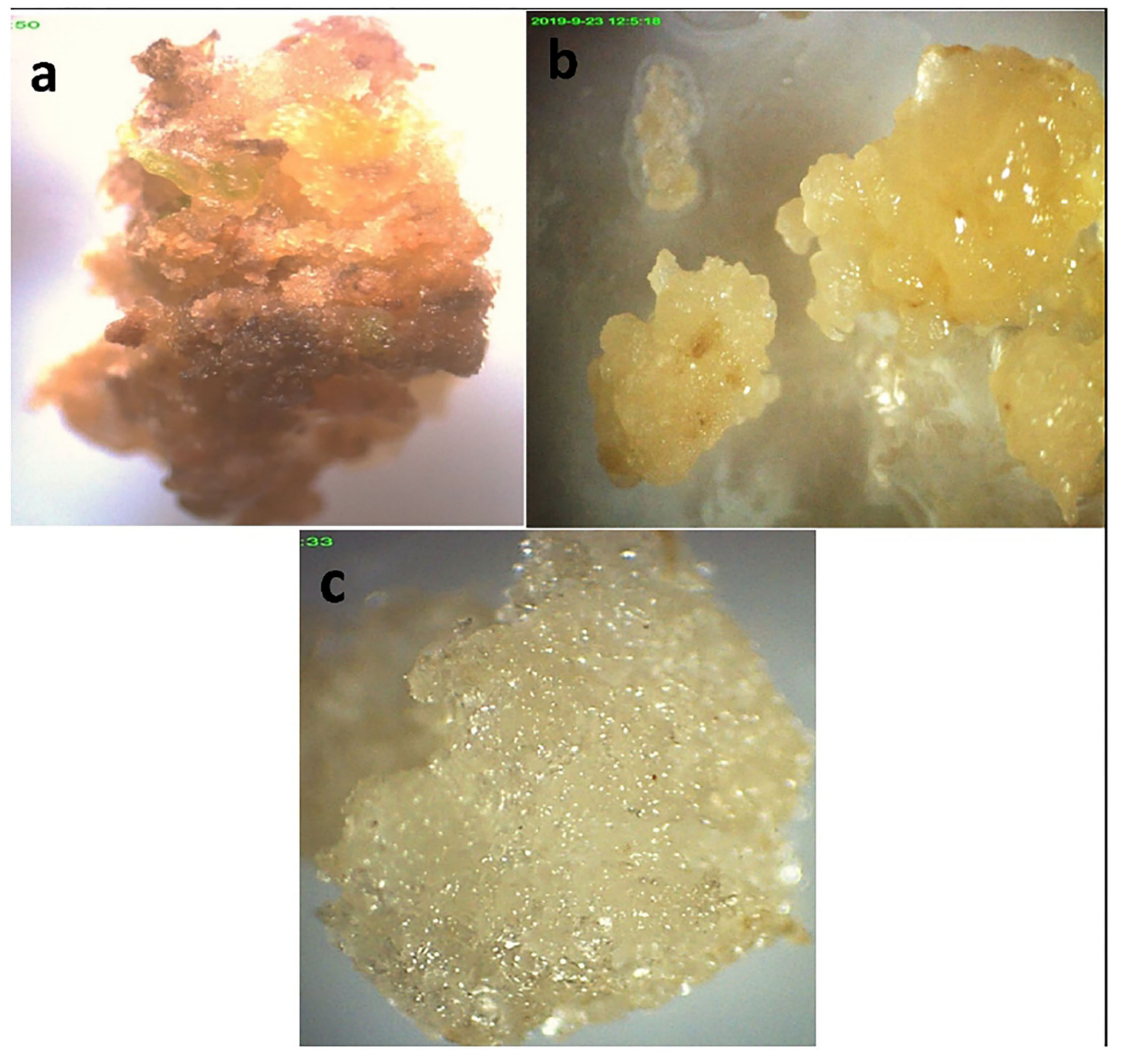
Callus culture establishment of three varieties of mung bean in MS + 2,4-D 3 mg/L **(a)** variety 1; **(b)** variety 3; **(c)** variety 2.

Different combinations of media and various concentrations were used for maximum callus induction. The results revealed that a high variation in callus induction and texture was established by varieties and growth regulators used. The highest callus induction (90.0 and 80%) was observed using leaf explants in media supplemented with MS + 3 mg/L 2,4-D (CT3) in Mung NCM-13, MgAT-7, and MgAT-4 varieties. The maximum weight of callus per explant (1.11 g) was obtained from variety 1 established on CT3. The addition of other phyto-regulators did not significantly affect the weight of the callus per explant and the texture of the callus (Figure 4 and Table 3). The addition of BAP to the medium along with 2,4-D made the callus compact and brown. Moreover, the addition of NAA also showed almost similar effects to the callus cultures of the three mung varieties.

**Table 3:** Effect of various concentrations and combinations of different growth regulators on callus cultures of three selected varieties of mung bean.

**Treatment #**	**MS Media supplemented with (mg/L)**	**Characteristics of callus**					
		**V1**		**V2**		**V3**	
		**Texture**	**Weight**	**Texture** **color**	**Weight**	**Texture** **color**	**Weight**
CT1	2, 4-D 1	compact, brown	0.24^c^ ± 0.02	Friable, green	0.19^b^ ± 0.02	Friable, Translucent	0.15^d^ ± 0.01
CT2	2, 4-D 2	compact, brown	0.47^b^ ± 0.04	Friable, brown	0.10^b^ ± 0.01	Friable, Translucent	0.64^b^ ± 0.03
CT3	2, 4-D 3	Friable, brown	1.11^a^ ± 0.04	Friable, translucent	0.64^a^ ± 0.03	Friable, brown	0.96^a^ ± 0.08
CT4	2, 4-D 1+ BAP 0.5	compact, brown	0.34^c^ ± 0.03	compact, brown	0.33^b^ ± 0.05	compact, brown	0.36^c^ ± 0.02
CT5	2, 4-D 2+ BAP 0.5	compact, brown	0.11^d^ ± 0.01	compact, brown	0.27^b^ ± 0.02	compact, brown	0.36^c^ ± 0.03
CT6	2, 4-D 3+ BAP 0.5	Friable green	0.70^b^ ± 0.03	compact, brown	0.15^b^ ± 0.01	Friable, brown	0.48^bc^ ± 0.03
CT7	2, 4-D 1+BAP 1	Dry friable	0.18^d^ ± 0.02	compact, brown	0.42^ab^ ± 0.03	Friable, Translucent	0.14^d^ ± 0.01
CT8	2, 4-D 2+ BAP 1	compact, opaque	0.06^d^ ± 0.01	compact, brown	0.13^b^ ± 0.01	compact, brown	0.26^c^ ± 0.05
CT9	2, 4-D 3+BAP 1	compact, opaque	0.11^d^ ± 0.01	friable, translucent	0.17^b^ ± 0.02	compact, brown	0.65^b^ ± 0.01
CT10	2, 4-D 1+ NAA 0.5	compact, brown	0.35^c^ ± 0.03	compact, brown	0.31^b^ ± 0.02	compact, brown	0.48^bc^ ± 0.02
CT11	2, 4-D 1+ NAA 1	compact, brown	0.10^d^ ± 0.01	compact, brown	0.44^ab^ ± 0.03	compact, brown	0.45^bc^ ± 0.03
CT12	2, 4-D 2+ NAA 0.5	compact, brown	0.21^c^ ± 0.02	compact, brown	0.21^b^ ± 0.01	compact, brown	0.22^c^ ± 0.02
CT13	2, 4-D 2 + NAA 1	compact, brown	0.58^b^ ± 0.01	compact, brown	0.15^b^ ± 0.01	compact, brown	0.12^d^ ± 0.01
CT14	BAP 0.5+ NAA 0.5	friable, granular	0.29^c^ ± 0.01	compact, brown	0.14^b^ ± 0.03	compact, brown	0.11^d^ ± 0.03
CT15	BAP 0.5+NAA1	compact, brown	0.36^c^ ± 0.03	compact, brown	0.18^b^ ± 0.03	compact, brown	0.19^d^ ± 0.02
CT16	BAP 1+ NAA 0.5	compact, brown	0.21^c^ ± 0.02	compact, brown	0.11^b^ ± 0.02	compact, brown	0.18^d^ ± 0.03
CT17	BAP 1+ NAA 1	compact, brown	0.69^b^ ± 0.06	compact, brown	0.12^b^ ± 0.01	compact, brown	0.06^e^ ± 0.02

### Phenolic Analysis of Shoot and Callus Cultures of Mung Bean

**Figure 5:**
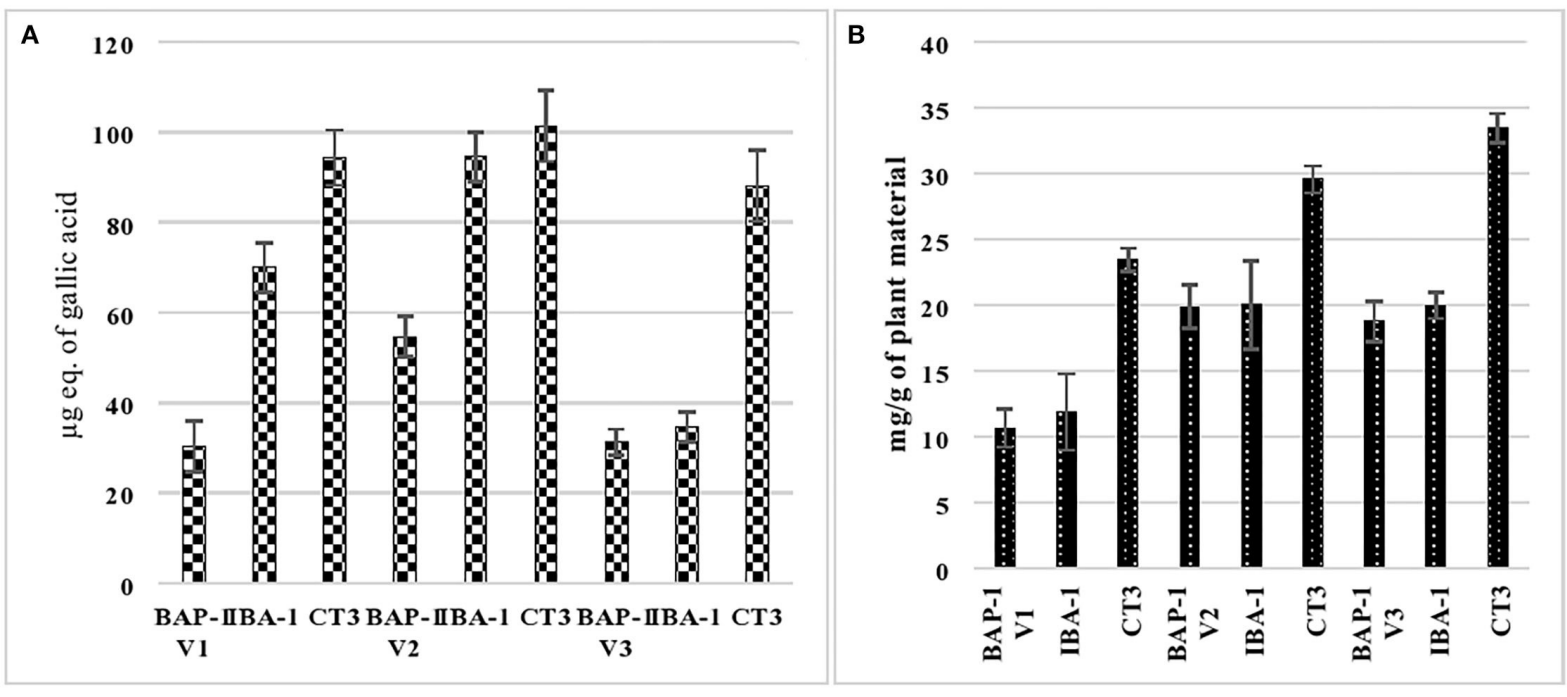
Phenolic **(A)** and glycoside **(B)** analysis of selected cultures of three varieties of mung bean.

Figure 5A shows the phenolic analysis of cultures of V1, V2, and V3 varieties from MS supplemented with 1 mg/L BAP, 1 mg/L IBA, and 3 mg/L 2,4-D. These cultures were selected based on their maximum growth response. It is clear from the figure that among all mung bean varieties, callus grown in MS+2,4D (3 mg/L) medium gave the highest amount of phenolics (30.33, 54.7, and 31.23 μg of GAE, respectively, for V1, V2, and V3 varieties). However, the cultures grown in MS+BAP (1 mg/L) supplemented medium showed minimum or least phenolic content (94.33, 101.45, and 88.1 μg of GAE, respectively, for V1, V2, and V3 varieties). The varietal difference can also be seen here in terms of phenolic content, as variety 2 (MgAT-7) showed maximum phenolic content in all the media used.

### Glycoside Analysis of Shoot and Callus Cultures of Mung Bean

Figure 5B indicates the glycosidic analysis of the selected cultures (as per Section Phenolic Analysis of Shoot and Callus Cultures of Mung Bean) of three mung bean varieties. It is clear from the figure that among all the mung bean varieties, callus cultures grown in MS+2,4-D (3 mg/L) produced higher amounts of total glycosides (23.46, 29.5, and 33.45 mg/g of plant tissue in V1, V2, and V3, respectively) when compared to other shoot cultures. Minimum glycoside content was found in cultures supplemented with the 1 mg/L BAP (20.87, 19.89, and 18.76 mg/g of plant tissue in V1, V2, and V3, respectively). The varietal difference can also be seen here in terms of glycoside content, as variety 3 (MgAT-4) showed maximum glycoside content in all the media used.

### Synthesis and Characterization of NPs

**Figure 6:**
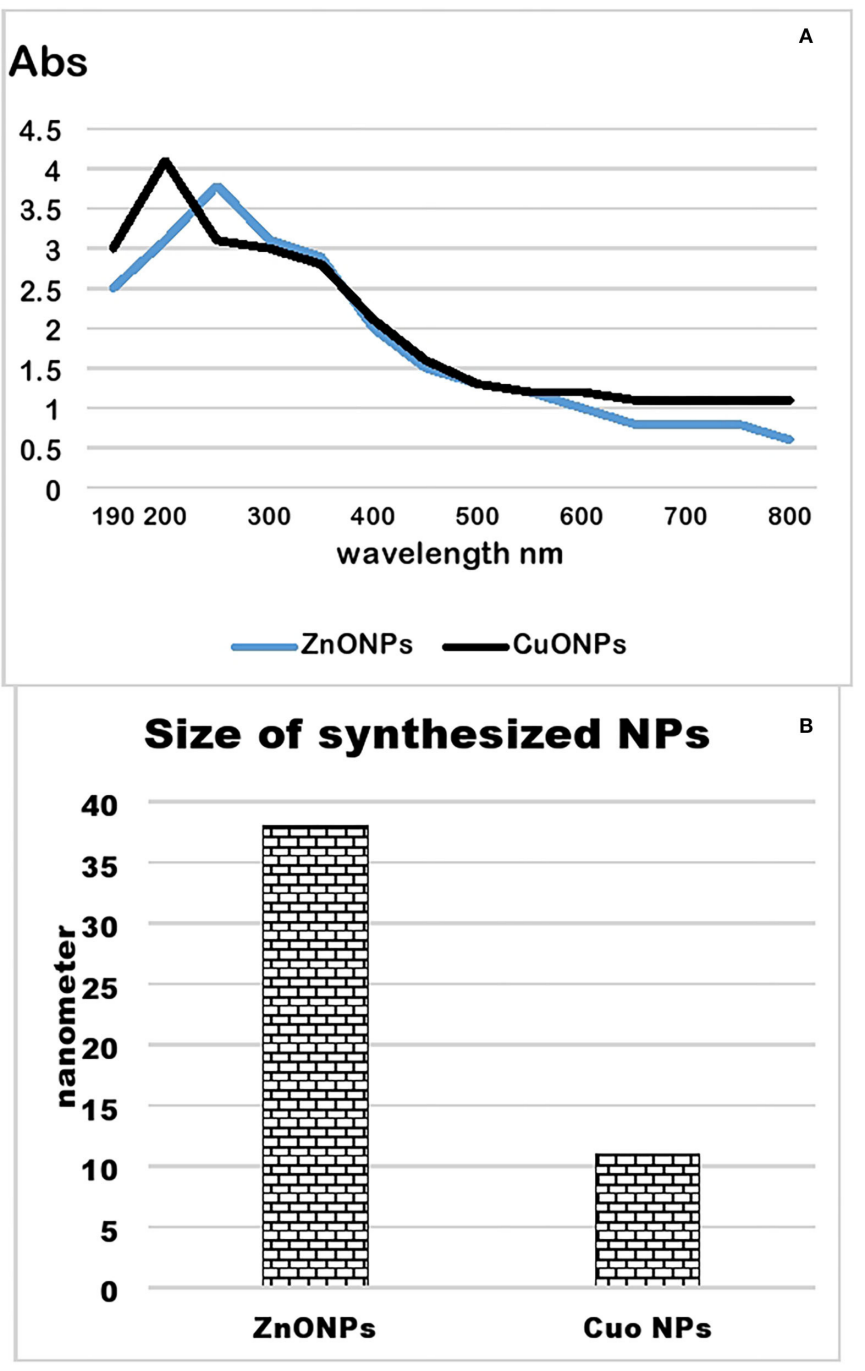
UV-visible spectrum **(A)** and particle size analysis **(B)** of zinc and copper oxide nanoparticles.

Zinc oxide and copper oxide nanoparticles were synthesized from the plant extract of *Nigella sativa*. A color change from brown to black indicated the synthesis of zinc oxide nanoparticles. Similarly, a color change from blue to light creamy brown indicated the synthesis of copper oxide nanoparticles. A UV-Visible spectrum of these NPs showed sharp peaks at 200 and 240 nm, respectively (Figure 6A). Particle size analysis of green synthesized zinc oxide and copper oxide nanoparticles is shown in Figure 6B. The results indicate an average size of 37.8 nm for zinc oxide nanoparticles and 11.5 nm for copper oxide nanoparticles.

### Zinc Oxide and Copper Oxide Nanoparticles as Elicitors

**Figure 7:**
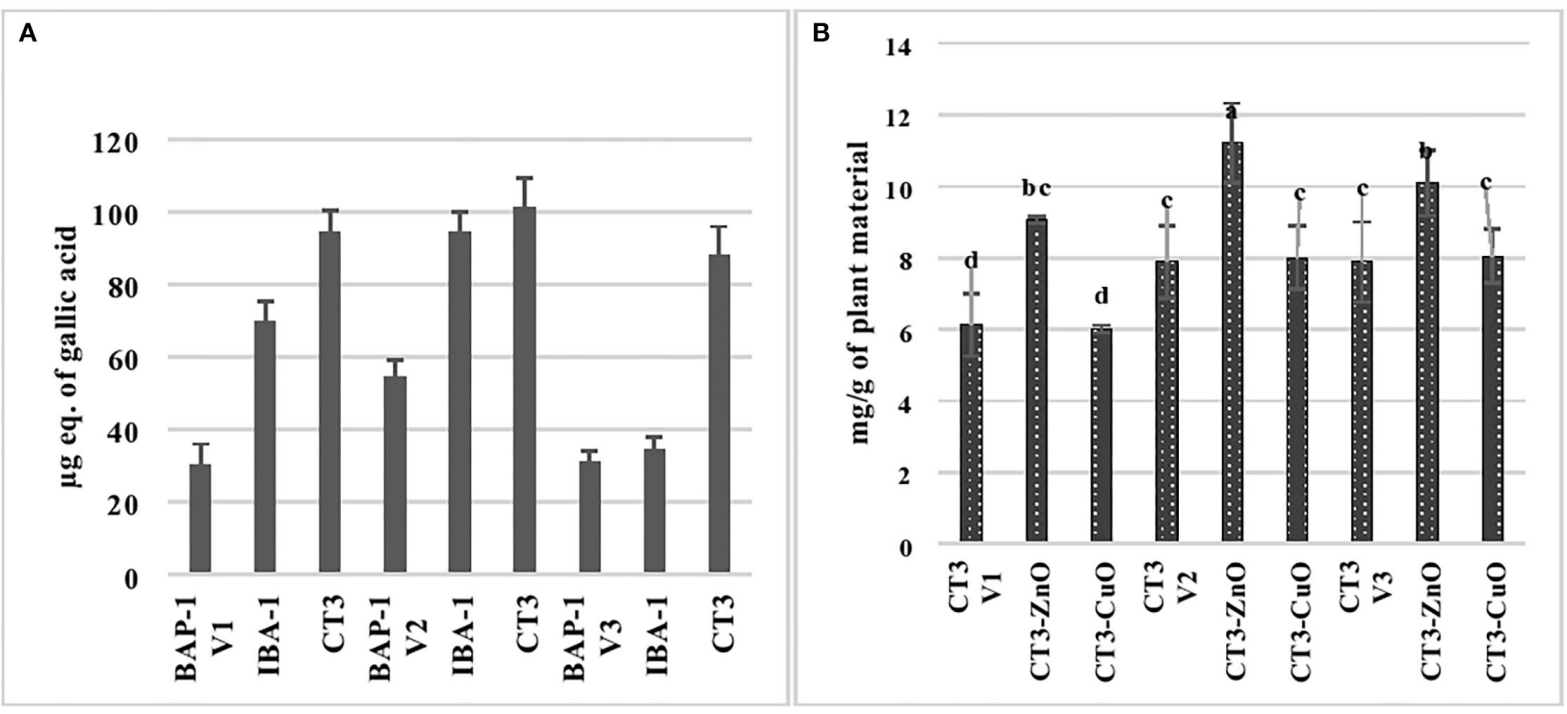
Phenolic **(A)** and glycoside **(B)** analysis of copper oxide and zinc oxide nanoparticles elicited cultures of three varieties of mung bean.

Figure 7A shows the effect of elicitors, i.e., zinc oxide and copper oxide nanoparticles, on phenolic production by callus cultures of three varieties of mung bean, and callus cultures grown in CT3 medium, i.e., MS+2,4-D (3 mg/L), were selected from Section Glycoside Analysis of Shoot and Callus Cultures of Mung Bean based on the findings that it produced maximum phenolics and glycosides. Results showed that ZnO NPs increased phenolic contents by 15, 19.5, and 16.2% in V1, V2, and V3, respectively. The introduction of copper nanoparticles to the CT3 medium increased phenolic content by 27, 23.8, and 19.95% in V1, V2, and V3, respectively. It is clear from the results that copper nanoparticles affected the phenolic content of all the three varieties of mung bean when compared to the zinc oxide nanoparticles. Figure 7B depicts the effect of elicitors, i.e., zinc oxide and copper oxide nanoparticles, on glycoside production by callus cultures of three varieties of mung bean, and callus cultures grown in CT3 medium, i.e., MS+2,4-D (3 mg/L), were selected from Section Glycoside Analysis of Shoot and Callus Cultures of Mung Bean based on the findings that it produced maximum phenolics and glycosides. Results showed that ZnO NPs increased glycosidic contents by 48, 42, and 27.8% in V1, V2, and V3, respectively, while the introduction of copper nanoparticles to the CT3 medium increased glycosidic content by 1.5 and 2 % in V2 and V3, respectively. However, copper oxide nanoparticles did not affect the glycosidic content of callus culture of the V1 variety of mung bean. It is clear from the results that zinc oxide nanoparticles affected the glycosidic content of all the three varieties of mung bean, whereas copper oxide nanoparticles did not show much efficacy for the above-said purpose.

## Discussion

In the current study, we deal with developing a protocol for the multiple shooting and callogenesis of *Vigna radiata* L. varieties in various concentrations of nutrient media and determining the effect of nano-elicitation on the secondary metabolite production of mung bean cultures. Results showed that the type of explant has a predominant effect on growth. Each explant has its own endogenous plant growth regulator (PGR) composition and morphological complexity, which in combination with the dose of external PGR plays its role in shoot and root initiation (Khawar et al., 2005). In the present study, shoot explant grown *in vitro* gave better response in shoot culture establishment. Earlier, researchers have reported a predominant effect of explant type on *in vitro* culture establishment (Hazra et al., 2000; Solorzano-Cascante et al., 2018).

The selection of a suitable explant is the first step for a successful experiment of plant tissue culture. Survivability of the culture is increased if the explant is selected from controlled conditions of a greenhouse (Kataky and Handique, 2010). This can be related to the results of the present study, where the selection of *in vitro* explants decreased the chances of contamination, which subsequently increased the overall response of mung bean cultures. Shoots and internodes are already considered as better explants (Dar et al., 2012). Similarly, Himabindu et al. (2014) developed an effective method to increase shoot multiplication in the green gram culture using cotyledonary nodes. However, the use of cotyledonary nodal fragments as explant is time-consuming and labor-intensive work that also requires specialization. In comparison, the present work reports the use of simple explants which is easy to use.

Plant growth regulators (PGRs) are organic substances that promote, inhibit, or alter plant growth and development in low concentrations (Yadav et al., 2010). In the present study, MS medium supplemented with cytokinin, i.e., 1 mg/L benzyl amino purine (BAP), gave the best results for the number of shoots and leaves per explant in all the three varieties of mung bean. A lot of work earlier has been presented on the positive influence of BAP on beans, although most of the legumes have shown recalcitrant behavior in tissue culture (Clapa, 2013; Mangena, 2020). Himabindu et al. (2014) developed an effective method to increase shoot multiplication in the green gram culture using Gamborg (B5) medium containing 0.5 mg/L BA. A moderate effect of BA in the production of multiple shoots has been previously reported in the mung bean by Vats et al. (2014). There are also some other reports of tissue culture of mung bean varieties where BAP produced better results (Hoque et al., 2007; Bhajan et al., 2019). Some *in vivo* studies also show that foliar application or spray of BAP gave higher per hectare yields of mung beans (Sarker et al., 2021).

The type of PGR has a significant effect on the branching and number of branches per explant. Cytokinins are usually involved in shoot formation, and the most commonly used is BAP (Herman, 2015; Phillips and Garda, 2019). BAP has various biological roles, such as it promotes adventitious shoot formation, cell division, flowering, and fruiting, and is known to retard apical dominance, leaf senescence, etc. The role of BAP in introducing lateral branches is also attributed to its capability of reducing apical dominance (Mangena, 2020). Selection of an appropriate concentration of selected plant growth regulator is also a very important factor, as too much higher or lower concentration of that particular growth regulator can cause the failure of tissue culture experimentation. In this regard, the type and variety of experimental plants also play a crucial role, as some plants have their endogenous cytokinin or auxin concentration, which is enough for their normal growth. In such cases, further application of exogenous plant growth regulators can have a negative effect (Phillips and Garda, 2019; Mangena, 2020; Fazeli-Nasab et al., 2021).

Furthermore, in the present study, the effect of various concentrations of IBA on root formation in *Vigna* species was also observed. Among the three *Vigna* species, a maximum root length of 5.2 cm and eight roots/explant were observed in V1 (Mung NCM-13) using MS + IBA (1.0 mg / L) medium when compared to other IBA concentrations. BAP-supplemented media was not able to induce rooting in mung bean cultures. It has previously been reported that BAP inhibits lateral root development and radicle development. Indole butyric acid (IBA) is a widely used PGR because it does not degrade quickly and is stable in culture media during autoclaving and storage (Costa et al., 2018). Earlier reports showed that in *Vigna radiata*, the adventitious root induction was seen after using the IBA, but not the IAA (Pan and Tian, 1999). In addition, IBA has also been reported to induce rooting response successfully in the cultures of Arabidopsis, blueberry, Hawthorn *Morus alba*, oak, orchids, etc. (Rafique et al., 2012; Guo et al., 2019; Motaghi and Mokhtari, 2019; Rafeeq et al., 2020).

Indole butyric acid promotes root growth by promoting cell expansion and proliferation (Iqbal et al., 2016). IBA has been known to change the expression of root-expressing genes, expression of auxin signaling pathway genes, and controls carbohydrate metabolism. They are known to accelerate the starch utilization in root cells (Xiao et al., 2020), thus increasing the root number and length. IBA is also known to induce a rooting response in explants by being converted into indole acetic acid (IAA), a natural auxin that is less stable in cultures when applied exogenously. In simple words, IBA can act as a precursor of IAA in explant cultures (Fattorini et al., 2017).

In our study, callus was developed using the immature 4–7-day old leaves of mung bean when cultured in MS medium supplied with different combinations of PGRs. Among these Vigna varieties, Mung NCM-13 showed maximum callus induction (80–90%) of soft and brown colored callus of 1.11 ± 0.04 g in MS+2,4-D (3 mg/L) media when compared to other treatments and combinations. Reducing the concentration of MS+2,4-D to 1.0 mg/L reduced the percentage of callus induction and gave the compact brown callus in the V1 variety. The addition of other phyto-regulators did not significantly affect the callus weight and texture in each explant. Various plant hormones have been widely used to establish the *in vitro* callogenesis of mung beans. Measuring the levels of auxin and cytokinin in the basal medium is very important because it leads to the differentiation pathways of the plant (Bourgaud et al., 2001). 2,4-Dichlorophenoxy acetic acid (2,4-D) is a commonly used auxin to increase callus growth because it can restore the cells to their original state and ability to divide (George et al., 2008).

Furthermore, a high endogenous level of auxins in explants can regulate cell division and growth (Tian et al., 2018). In mung beans, for callus induction, the value of 2, 4-D was studied by Amutha et al. (2003). Previously, Rao et al. (2005) studied the effect of two synthetic auxins, namely, 2, 4-D and NAA, on the initiation of callus in mung bean varieties. Low concentrations of 2,4-D and NAA did not support callus growth, but high concentrations of 2,4-D showed a better effect when compared to NAA. Similarly, Patra et al. (2018) reported high variability in callus induction, shoot, and root regeneration. About 90–100% of nodal explants of green gram cv. IPM-02-03 initiated calli when grown in an MS medium modified using different concentrations of BAP and NAA.

Further, in the present study, phenolic and glycosidic analysis of various cultures (shoot cultures, rooted shoot cultures, and callus cultures for V1, V2, and V3) was carried out, which showed that callus cultures had the highest biochemical content. Three varieties of mung bean showed the same trend, showing higher phenolic and glycosidic content in callus cultures when compared to root and shoot cultures. However, the differences between the varieties can be attributed to their genetic makeup or simply varietal differences. Earlier, Janarthanam et al. (2010) reported higher stevioside content (a glycoside) in callus cultures as compared to leaves of the mother plant. Palacio et al. (2012) also reported significant phenolic content in the callus cultures of *Larrea divaricata*, which is comparable to intact plants. Nadia et al. (2020) revealed higher content of phenolic compounds in the callus cultures of maize supplemented with 2,4-D when compared to enrichment with other PGRs. Zaman et al. (2021) reported higher alkaloid content in the callus cultures supplemented with 2,4-D. Phenolic compounds are important metabolites in plants and are undoubtedly the major phytochemicals targeted for medicinal purposes. Phenolic acids have been associated with several health functions, such as anticancer (Salehi et al., 2019) and antidiabetic (Swieca et al., 2014) activities and many other biological functions in humans. Glycosides are also known for their health benefits, including antioxidant, anticancer, hepatoprotective, antidiabetic, anti-inflammatory, antipyretic, memory enhancing, antiviral, antibacterial, antifungal, and antiaging activities (Xiao et al., 2016). Enhanced production of both these components has great commercial importance.

Plant tissue culture and its related extensions are considered a strong tool for the sustainable production of secondary metabolites of commercial importance (Espinosa-Leal et al., 2018). The extraction of plant-specific bioactive compounds like phenolics, alkaloids, and glycosides from the wild plant is time-consuming with low extraction yields. Also, the yield of plant secondary metabolites from specific plant parts depends on the growth and developmental stages, season, and stress type, as well as nutrient availability (Ramirez-Estrada et al., 2016). In the same way, with the increasing demand for food and related products, food crops cannot be replaced by other commercial crops for secondary metabolite production on available arable land. Therefore, manipulation of secondary metabolite production in plant cell and tissue culture by varying their PGR type and concentration is a better and sustainable choice (Palacio et al., 2012). Plants produce secondary metabolites in response to stress. PGRs create oxidative stress in plants, which gives rise to reactive oxygen species (ROS). These ROS then activate the signaling pathway to produce secondary metabolites like phenolics, alkaloids, etc. (McCarthy-Suárez, 2017), since callus cells are totipotent and have a complete genetic makeup to synthesize all plant metabolites.

In the present study, nano-elicitors, including zinc oxide (ZnO) and copper oxide (CuO) nanoparticles (NPs), have significantly improved the total amount of phytochemicals (glycosidic and phenolic content) in the callus cultures of mung bean. Callus cultures were selected for elicitation. as they gave higher content of phenolics and glycosides when compared to shoot cultures (Figures 5A,B). CuO NPs increased the phenolic content of callus cultures of mung bean varieties, whereas ZnO NPs enhanced the glycosidic content of the callus cultures.

Elicitors have been widely used to enhance the production of secondary metabolites in the cells of plant and organ cultures. The effects of various abiotic and biotic elicitors on the production of secondary metabolites in plant tissue cultures depend on specific plant varieties and the type of targeted secondary metabolites. Elicitor concentrations play a very important role in the elicitation process. It was reported that high doses of elicitor produced a hypersensitive response leading to cell death, and an optimum dose was required for induction (Aloo et al., 2021). Here, the role of green synthesized nanoparticles as elicitors has been proven to produce positive results. Callus cultures and transformed hairy root cultures are already being used to get an enhanced quantity of pure and specific biologically active compounds from various plants (Gabr et al., 2019; Hussain et al., 2022). According to some researchers, additional use of nanoparticles is just helpful to develop new original methods of getting a high level of specific secondary metabolites. Green synthesized nanoparticles have proven their significance due to their biocompatibility and stability in plant tissue cultures. NPs are also considered to be more active, more effective, and superior in behavior when compared to their macro-size salts due to their nano dimensions and larger surface area (Iravani, 2011; Karimi et al., 2018), and these characteristics increase their efficiency. Nanoparticles as elicitors can play a dual role, the first is by acting as an efficient nutrient and the second is by acting as an elicitor to enhance the secondary metabolite production.

Our results are in line with Zaeem et al. (2020) and Bhardwaj et al. (2018), who reported higher production of phenols in calli supplemented with zinc oxide nanoparticles. Similarly, ZnO NPs have been reported to increase the flavonoid and phenolic content in the *Glycyrrhiza glabra* plants (Oloumi et al., 2015). *Stevia rebaudiana* callus was treated with ZnO NPs, which showed an increase in phenolic and flavonoid content. ZnO NPs produce active forms of oxygen species that enhance antioxidant responses, and the production of secondary metabolites is greatly enhanced (Javed et al., 2018; Ahmad et al., 2020). Other studies have reported that abiotic stress causes an increase in the production of the free amount of phenolic content in legume sprouts (Limón et al., 2014; Swieca et al., 2014).

Copper oxide NPs have been proposed as an elicitor of important bioactive compounds in the bioreactor system (Singh and Dwivedi, 2018). Zhao et al. (2017) reported the effects of Cu NPs on cucumber fruits using different concentrations as elicitors. As a result, they obtained increased levels of essential amino acids, such as valine, leucine, isoleucine, threonine, and tyrosine. NPs as elicitors bind on the cell membrane and activate mitogen-activated protein kinase proteins and ion channels, which results in the production of ROS species, producing more secondary metabolites like glycosides in mung beans.

## Conclusion

In conclusion, all three varieties of *Vigna radiata* L. showed higher phytochemical content in the callus cultures grown in MS medium supplemented with 2,4-D (3 mg/L). ZnO NPs and CuO NPs were used as nano-elicitors in callus cultures, where they significantly enhanced the glycoside and phenolic content of the cultures, respectively. Improved glycoside production in the *in vitro* cultures of mung beans may be achieved by further modulating the *in vitro* cultures, the type, and concentrations of PGRs and nanoparticles, as mung bean cultures contain amygdalin, a glycoside of great commercial importance due to its anticancer activity. In continuation of this research work in the future, specific types of phenolics and glycosides would be determined using HPLC techniques.

## Data Availability Statement

The original contributions presented in the study are included in the article/supplementary material, further inquiries can be directed to the corresponding authors.

## Author Contributions

ZI: experimentation. SJ: visualization and supervision. SN: validation and research design. AAS: review and drafting. ANS: data analysis. BP: statistical analysis and manuscript drafting. AG: manuscript drafting and analysis. NA: research idea and design. All authors contributed to the article and approved the submitted version.

## Funding

This study was supported by the Researchers Supporting Project Number RSP-2021/144, King Saud University, Riyadh, Saudi Arabia.

## Conflict of Interest

The authors declare that the research was conducted in the absence of any commercial or financial relationships that could be construed as a potential conflict of interest.

## Publisher's Note

All claims expressed in this article are solely those of the authors and do not necessarily represent those of their affiliated organizations, or those of the publisher, the editors and the reviewers. Any product that may be evaluated in this article, or claim that may be made by its manufacturer, is not guaranteed or endorsed by the publisher.
